# Mental Nerve Paraesthesia: A Report of Two Cases Associated with Endodontic Etiology

**DOI:** 10.1155/2021/1747519

**Published:** 2021-10-13

**Authors:** Neeta Patel, Akshayraj Langaliya, Shikha Kanodia, Aravind Kumbhar, Aastha Buch, Aarshvi Shah, Himani Bhatt, Drashti Panchal, Sharan Shah, Jinali Shah

**Affiliations:** ^1^AMC Dental College and Hosptial, Bhalakhiya Mill Compound, Opp. Anupam Cinema, Ahmedabad 380008, India; ^2^Government Dental College and Hospital, Civil Hospital Campus, Asarwa, Ahmedabad 380016, India; ^3^Faculty of Dental Sciences, Dharmsinh Desai University, Nadiad 387001, India

## Abstract

Paraesthesia of the mental nerve can occur due to various etiological factors. Rarely, dental infections can cause paraesthesia. However, this article discusses two cases of endodontic etiology in the mental nerve region as a causative factor for paraesthesia. In the first case, the patient had severe pain localized to his right mandible, with numbness of his lower lip. Endodontic treatment led to quick regression and resolution of paraesthesia. In the second case, a patient who was referred for retreatment of a mandibular second premolar infection developed profound paraesthesia in the region of the mental nerve distribution following prior therapy. Possible mechanisms responsible for periapical infection-related paraesthesia are discussed here. CBCT imaging may be useful in the diagnosis and management of such conditions.

## 1. Introduction

Paraesthesia is a feeling that is rare, provocative, or spontaneous and is commonly described by patients as a burning, tickling, or tingling sensation [1]. According to the taxonomy of the International Association of Study of Pain (IASP), paraesthesia, although abnormal, is not unpleasant [2]. Patients have often described it as warmth, cold, burning, aching, prickling, tingling, pin and needle sensation, or numbness.

The mental foramen in dentistry is an essential anatomical feature on the lateral surface of the mandible. This marks the culmination of the mandibular canal that opens in an oblique direction to the surface. The mental nerve bundle passes through the mental foramen and supplies the soft tissues of the chin, lower lip, and gingiva on the ipsilateral side of the mandible with sensory innervation and nutrition [3].

Radiographically, the mental foramen may result in misdiagnosis of a radiolucent lesion in the apical area of the mandibular premolar teeth. For diagnostic and clinical procedures, accurately locating the mental foramen is important [4]. The intimate anatomical proximity between the root apices of the mandibular premolars and the mental nervous bundle should also be meticulously examined. Periapical infection before and after endodontic therapy leading to mental and inferior alveolar nerve paraesthesia is also well documented [5]. The mechanism of induction of paraesthesia due to periapical infection may be threefold.

1. The inflammatory process causes purulent exudate to accumulate in the mandibular bone, causing local mechanical pressure on the mental nerve [6, 7]
2. Toxic or inflammatory bacterial products [6–8]
3. Adequate subsequent hematoma pressure [6, 7]
4. Direct invasion of nerve structures by bacteria themselves [9]

## 2. Case Reports

### 2.1. Case 1

**Figure 1:**
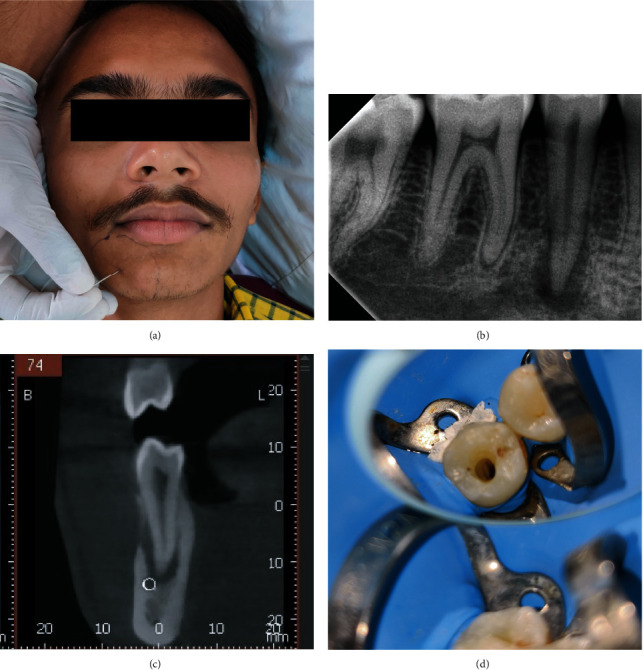
(a) Paraesthesia testing, (b) preoperative RVG, (c) preoperative CBCT, and (d) access cavity under dental dam.

A 21-year-old healthy male patient reported to our institute with severe pain localized to his right mandible and numbness on the right side of the lower lip. On examination, the patient was apyrexic with normal vital signs, no apparent facial asymmetry, and a 42 mm incisal mouth opening without pain. The patient had a trauma history on the same side 1 month prior. No carious lesion was evident on clinical assessment. The lower right second premolar was tender on percussion. Electric pulp testing revealed an intact sensation in the mandibular right first molar and right first premolar but no response in the mandibular right second premolar. Two-point discrimination light-touch and pin-prick tests were performed on the right mentum region encompassing the area of the skin below the right half of the lower lip and anterior part of the cheek on the same side, which confirmed persistent paraesthesia (Figure 1(a)). A RadioVisio Graphic (RVG) image revealed radiolucency in the apical region involving the mental foramen (Figure 1(b)). To further confirm the diagnosis, a cone-beam computed tomography (CBCT) scan (Vatech PaX I 3D Smart, 74 kV, 10 mA, 10.8 sec) of the same region revealed a large periapical radiolucency involving the second premolar extending into the mental foramen (Figure 1(c)). Nonsurgical endodontic treatment was planned. Informed consent was obtained before initiation of treatment.

**Figure 2:**
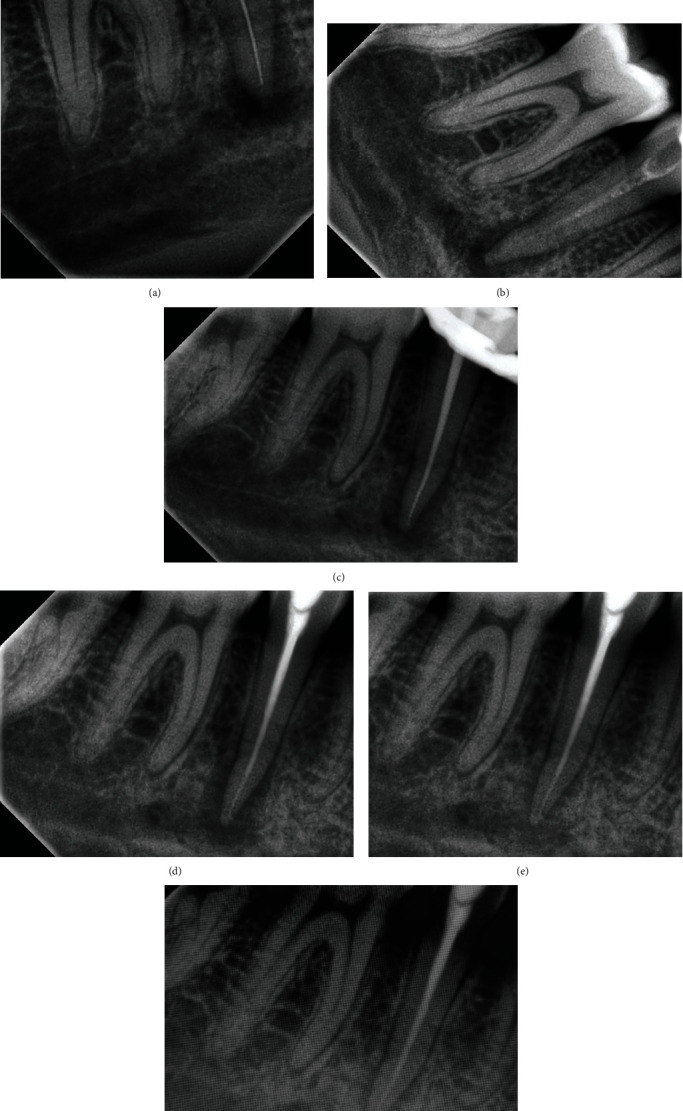
(a) Working length determination, (b) calcium hydroxide placement, (c) master cone selection, (d) obturation, (e) 3-month follow-up, and (f) 6-month follow-up.

Root canal treatment was initiated under isolation with a dental dam (Figure 1(d)). Patency filing was performed with a stainless steel 10# K file (Dentsply-Maillefer, Switzerland). The working length was determined using an electronic apex locator (Root ZX mini, Morita, Osaka, Japan), which was later confirmed by the RVG image (Figure 2(a)). Cleaning and shaping were performed by Protaper Gold files (Dentsply-Maillefer, Switzerland) until F2 size with careful intermittent copious irrigation with 3% sodium hypochlorite (Vishal Dentcare, Ahmedabad, India) solution followed by normal saline and a final rinse of 17% EDTA (Prime Dental Products, Thane, India). The canal was carefully dried with paper points, and an intracanal medicament of calcium hydroxide with a mixture of 2% CHX (Vishal Dentcare, Ahmedabad India) was placed (Figure 2(b)). The tooth was restored with an interim restoration (Cavit 3M, USA), and the patient was discharged with oral antibiotics Tab. Amoxycillin-Clavulanic acid–625 mg twice daily for seven days and vitamin B oral supplements.

The patient was recalled after 7 days and evaluated for symptoms of paraesthesia. There seemed to be a mild recovery from numbness in the chin region and complete recovery from pain and tenderness. Two weeks later, the paraesthesia had almost disappeared except for a small patch on the right side of the lower lip. The tooth was completely asymptomatic; therefore, obturation was performed at this visit with laterally compacted gutta-percha with MTA-based sealer (MTA Plus, Prevest Denpro, India) (Figure 2(d)). At three- and six-month subsequent follow-ups, examination showed reconstitution of the apical region, cortical lining of the inferior alveolar canal, and paraesthesia was completely resolved (Figures 2(e) and 2(f)).

### 2.2. Case 2

**Figure 3:**
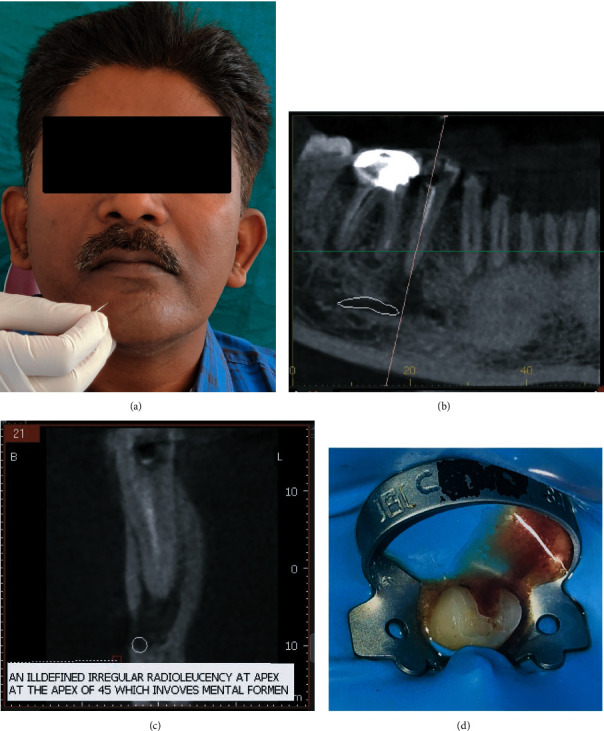
(a) Paraesthesia testing, (b, c) preoperative CBCT, and (d) access cavity under rubber dam isolation.

A 45-year-old male reported to our institute with pain, swelling, and tingling sensations in the lower right second premolar region. On examination, the patient was apyrexic with normal vital signs and had adequate mouth opening. The patient had previously undergone root canal treatment in tooth #45 before one year by a general dentist. The patient described that almost after 6 months of the initial treatment, he had an episode of sudden pain in relation to the same tooth but he took over-the-counter medications and the pain soon subsided. The next episode was a drastic one as described above which urged him to visit our department. On palpation, the right second premolar was tender, and two-point discrimination, light-touch, and pin-prick tests were performed as the above-mentioned case, which confirmed persistent paraesthesia (Figure 3(a)). To confirm the diagnosis of mental nerve involvement, CBCT was directly performed at the insistence of the patient. A small field of view (FOV) CBCT (Vatech PaX I 3D Smart, 74 kV, 10 mA, 10.8 sec) was obtained, which apparently depicted mental foramen involvement and improper root canal filling, necessitating retreatment in relation to the right second premolar (Figures 3(b) and 3(c)). Informed consent was obtained before beginning the treatment, and all possible outcomes were explained.

Under dental dam isolation, the previous restoration was removed, and gutta-percha was removed using a combination of H files (Dentsply Maillefer, Switzerland), ProTaper retreatment files D1 and D2 (Dentsply Maillefer, Switzerland), and gutta-percha solvent (Carvene Prevest Denpro, India) (Figure 3(d)). Intermittent copious irrigation was performed with 3% NaOCl followed by a final rinse with generically prepared 7% maleic acid and 2% CHX (Vishal Dentcare, Ahmedabad, India). An intracanal medicament of injectable calcium hydroxide (RC cal, Prime Dental Products, Thane, India) dressing was given in interim appointments (twice for three weeks), allowing the patient to be discharged with Tab. Amoxycillin-Clavulanic acid–625 mg twice daily for seven days and vitamin B oral supplements.

**Figure 4:**
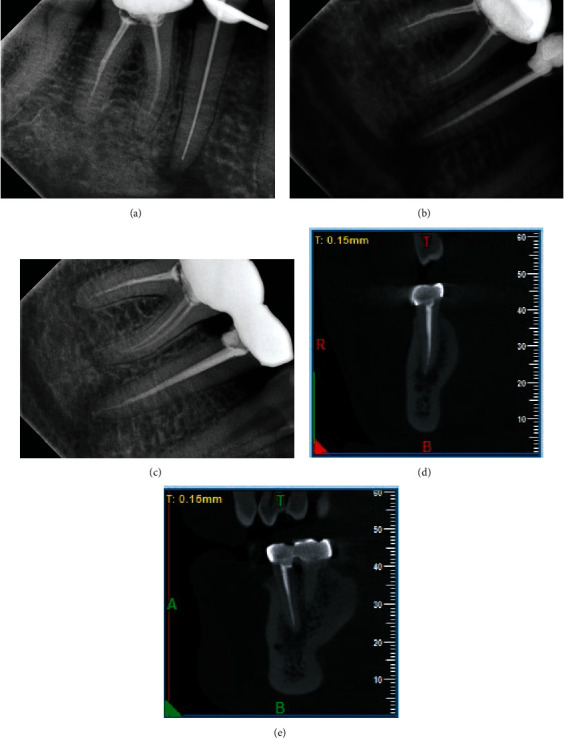
(a) Working length determination, (b) obturation, (c) 3-month follow-up RVG, and (d, e) 12-month follow-up CBCT.

The pain completely resolved within a span of three days, and a gradual reduction in paraesthesia occurred over a period of one and a half months. Subsequently, obturation was performed using the lateral condensation technique with an Endosequence Bioceramic sealer (Brasseler, Endo, USA). Three- and twelve-month follow-up examinations showed reconstitution of the apical region, labial cortex, and cortical lining of the mental foramen, and paraesthesia was completely resolved (Figures 4(c)–4(e)).

## 3. Discussion

Paraesthesia due to endodontic etiology can be attributed to various factors such as periapical infection in relation to an affected premolar. Localized pressure on the mental nerve due to an acute inflammatory response can cause Mental Nerve Paraesthesia (MNP). Gilbert and Dickerson reported a case of asymptomatic apical periodontitis that had undergone acute exacerbation leading to paraesthesia of the area supplied by the mental nerve [8]. It was found that the patient's use of home remedies and local aspirin tablets caused burns in the mental region and may have exacerbated the problem.

In the cases enumerated here, the causes of paraesthesia prior to and after previous endodontic treatment were carefully evaluated and individually considered in an attempt to establish the exact etiology. The patient was asked additional questions regarding the presence of other symptoms of neuropathy, such as numbness or altered sensation, and chair-side examination was performed [10]. Flare-up-related paraesthesia was ruled out due to a lack of other characteristic signs of infection [11].

CBCT offers a small field of view with relatively less radiation and is recommended as a diagnostic aid in examining causes, suggesting a safety zone for any procedure, and delineating important structures missed with conventional periapical radiography. There were no extra canals visible in CBCT imaging [12].

Paraesthesia due to direct injection of local anaesthetic solutions into the nerve or needle injury is common. Trauma caused by the contact of the needle with the nerve is probably related to an improper or misguided anaesthetic technique, causing haemorrhage that may lead to compression of the nerve and then paraesthesia. The hydrostatic pressure of the injection and mechanical nerve compression cause a reduction in blood flow to the nerve and its deformation, subsequently causing paraesthesia [13]. Inferior alveolar nerve paraesthesia is common due to local anaesthesia, but MNP rarely occurs due to the anaesthetic technique. Flanagan reported a case in which needle injury caused a hematoma in the mental region and compression of the mental nerve occurred, resulting in MNP [14]. Our cases did not warrant administration of local anaesthetic; hence, the possibility of this etiology was eliminated.

Extrusion of sodium hypochlorite is another factor to be considered. It is known that sodium hypochlorite when comes in contact with vital tissue causes haemolysis, ulceration, preventing neutrophil migration leading to injury to endothelial and fibroblast cells. The immediate sequel of such accidents includes acute sudden excruciating pain and an immediate swelling in the affected area. In severe cases, a necrotic ulceration of the tissue adjacent to the tooth usually follows which may appear immediately or a few days to weeks later. However, no such symptoms were observed in either of the cases and hence extrusion of the irrigant cannot consider a likely cause [15]. Consideration and careful attention were given to maintain the working length within the confines of the root. Overinstrumentation, causing trauma to the neurovascular bundle is unlikely in this case [16].

Several cases have been documented suggesting paraesthesia can be induced by extrusion of different sealers. Although sealers are biocompatible and nontoxic, some studies also indicate that endodontic materials can be neurotoxic at some level, initiating a host-dependent inflammatory process, which causes cellular damage, ulceration, and haemolysis when in contact with vital tissue. Some cases of MNP are reported due to the overfilling of sealer paste in the vicinity of the mental nerve.

Gutta-percha is considered a biologically inert material. The collective effect of overinstrumentation and excessive vertical pressure during compaction of the filling material cause extrusion of the filling material, and paraesthesia usually results from the overfilling of gutta-percha [17, 18].

Additionally, continuous wave of the condensation technique could possibly produce higher temperatures; hence, in these cases, we performed the cold lateral condensation technique, which was associated with a lack of bone necrosis or symptoms due to overheating. Meticulous attention to detail was exercised with regard to sealer placement and apical tug back of gutta-percha to avoid any overextension into the periapical area [19].

Vitamin B complex is the key pharmacological choice. The B complex used in this case was based on its safety profile and its potential to support recovery from nerve damage. Early treatment and prevention of periapical lesions by regular dental care is the most important and effective way to prevent MNP.

## 4. Conclusion

Two cases presented with periapical infection in the second premolar, which led to MNP. The most important part of managing such a condition associated with endodontic involvement is accurate diagnosis and/or prevention of iatrogenic errors. CBCT imagery strengthens our understanding of anatomy in this respect and may offer additional support. Elimination of endodontic etiology through conventional nonsurgical endodontic therapy in conjunction with vitamin B supplements led to successful and complete elimination of paraesthesia in due course of time.

## Data Availability

Any data related to the case report can be readily provided on reasonable request.
